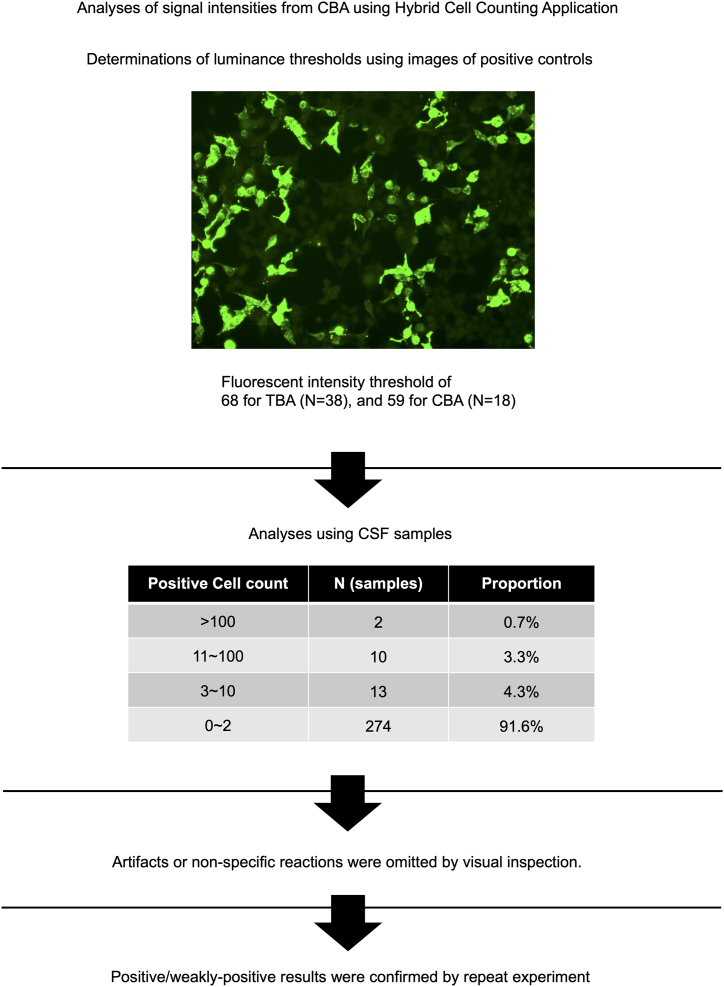# Neuronal autoantibodies in the cerebrospinal fluid of 148 patients with schizophrenia and 151 healthy controls

**DOI:** 10.1016/j.heliyon.2024.e30695

**Published:** 2024-05-05

**Authors:** Takako Enokida, Nanako Yoshida, Megumi Tatsumi, Shinsuke Hidese, Yu-ichi Goto, Mikio Hoshino, Hiroshi Kunugi, Kotaro Hattori

**Affiliations:** a Department of Bioresources, Medical Genome Center, National Center of Neurology and Psychiatry, 4-1-1, Ogawahigashi, Kodaira, Tokyo, 187-8502, Japan; b Department of NCNP Brain Physiology and Pathology, Cognitive and Behavioral Medicine, Graduate School of Medical and Dental Sciences, Tokyo Medical and Dental University, 1-5-45 Yushima, Bunkyo-ku, Tokyo, 113-8510, Japan; c Department of Psychiatry, Teikyo University School of Medicine, 2-11-1, Kaga, Itabashi-Ku, Tokyo, 173-8605, Japan; d Department of Mental Disorder Research, National Institute of Neuroscience, National Center of Neurology and Psychiatry, 4-1-1, Ogawahigashi, Kodaira, Tokyo, 187-8502, Japan; e Department of Biochemistry and Cellular Biology, National Institute of Neuroscience, National Center of Neurology and Psychiatry, 4-1-1, Ogawahigashi, Kodaira, Tokyo, 187-8502, Japan

**Keywords:** Autoantibody, Cerebrospinal fluid, NMDA, DFS70, Schizophrenia

## Abstract

Schizophrenia is a syndrome with multiple etiologies, one of which is the potential for an autoimmune disease of the brain such as N-methyl-d-aspartate receptor (NMDAR) encephalitis, which can induce psychosis resembling schizophrenia. Here, we examined anti-neuronal autoantibodies related to psychosis using both cell- (CBA) and tissue-based assays (TBA) in the cerebrospinal fluid (CSF) of patients with chronic schizophrenia and control participants. First, we screened for the antibodies against leucine-rich glioma-inactivated 1 (LGI1), γ-aminobutyric acid B receptor (GABABR), dipeptidyl aminopeptidase-like protein 6 (DPPX), α-amino-3-hydroxy-5-methyl-4-isoxazolepropionic acid receptor (AMPAR1/R2), and contactin-associated protein-like 2 (CASPR2) in 148 patients with schizophrenia. No antibodies were detected. Next, we performed CBA for NMDAR antibodies in 148 patients with schizophrenia and 151 age- and sex-matched controls. Although we detected relatively weak immunoreactivity for NMDAR in the CSFs of two patients with schizophrenia and three controls, no samples were positive when strict criteria were applied. For TBA in the rat hippocampus and cerebellum, we detected positive signals in the CSFs of 13 patients with schizophrenia and eight controls. Positive samples were analyzed for paraneoplastic syndrome and antinuclear antibodies using immunoblotting. The CSFs of nine patients and six controls were positive for dense fine speckle 70 (DFS70) antibodies. Additionally, antibodies against centromere protein (CENP)-A and CENP-B were detected in patients with schizophrenia. Our results suggest that autoantibodies against NMDAR, LG1, GABABR, DPPX, AMPAR1/R2, and CASPR2 are not associated with the pathogenesis of chronic schizophrenia. Moreover, we emphasize the importance of considering the effect of anti-DFS70 antibodies when analyzing autoantibodies in CSF samples. Conclusively, we obtained no evidence suggesting that the most frequent neuronal autoantibodies in the CSF play a role in the pathogenesis of schizophrenia, even in our sample.

## Highlights

• CSFs of 148 patients were negative for both NMDAR and most frequent PNS antibodies.
• CSFs of 13/148 patients and 8/150 controls were immunoreactive with rodent brain.
• 9/13 patients and 6/8 controls were DFS70 antibody-positive, not pathogenic.

## 1 Introduction

Schizophrenia is a progressive and chronic psychiatric disorder affecting approximately 0.3 % of people worldwide [1,2]. In recent years, schizophrenia has been considered a heterogeneous condition with differential etiological factors [3], such as neuroinflammation [4], genetic abnormalities [5], and autoimmune disorders [6,7].

Anti-N-methyl-d-aspartate receptor (NMDAR) antibody encephalitis is an autoimmune encephalitis mediated by antibodies against the glutamate receptor GluN1 subunit. Approximately 90 % of the patients have prominent psychiatric or behavioral symptoms at onset [8], making it difficult to differentiate them from psychiatric disorders, particularly schizophrenia [[9], [10], [11], [12]]. Other types of autoantibody encephalitis, such as paraneoplastic syndrome (PNS), are also known to cause psychiatric symptoms [13,14].

Cerebrospinal fluid (CSF) is a body fluid in close contact with the brain. The CSF contains molecules derived from neurons and glial cells and is useful in the search for biomarkers of brain disorders [15,16]. Blood samples are typically used for the detection of autoantibodies in patients with autoimmune disorders. However, in anti-NMDAR encephalitis, the CSF has a higher sensitivity to anti-NMDAR antibodies than blood [17]. Only approximately 85 % of the anti-NMDAR antibodies detectable in the CSF are detected in the serum, and a considerable rate of false positives has been suggested in serum testing [[18], [19], [20]].

In this study, we aimed to determine whether the most frequent neuronal antibodies associated with autoimmune encephalitis are involved in chronic schizophrenia. We analyzed the CSF from participants with schizophrenia and healthy controls and evaluated anti-neuronal antibodies, including NMDAR, α-amino-3-hydroxy-5-methyl-4-isoxazolepropionic acid receptor (AMPAR), leucine-rich glioma-inactivated 1 (LGI1), dipeptidyl aminopeptidase-like protein 6 (DPPX), contactin-associated protein-like 2 (Caspr2), and γ-aminobutyric acid B receptor (GABABR) via cell-based assay (CBA) and other autoantibodies via tissue-based assay (TBA) using rodent brain.

## 2 Materials and methods

### 2.1 CSF samples

CSF samples were obtained from the National Center of Neurology and Psychiatry (NCNP) Biobank, a member of the National Center Biobank Network [21]. Participants were recruited at the NCNP Hospital in Tokyo, Japan, or through website announcements. For participants with schizophrenia, a consensus diagnosis was made according to the DSM-IV criteria [22], and symptoms were assessed using the Japanese version of the Positive and Negative Syndrome Scale (PANSS) [23]. The total PANSS score was calculated by summing the scores on the positive, negative, and general psychopathology scales. The control participants were screened for psychiatric disorders using the Japanese version of the Mini-International Neuropsychiatric Interview (MINI) [24,25]. Daily doses of antipsychotics were converted to chlorpromazine (CP)-equivalent doses, according to published guidelines [26]. Disease duration was defined as the period from initial onset to CSF collection. Participants with a history of central nervous system disease, severe head injury, or substance abuse were excluded. CSF samples were obtained by lumbar puncture as described previously [27]. Written informed consent was obtained from all participants for lumbar puncture and biobanking of samples. For participants under the age of 20, informed assent was obtained from the individual after which written informed consent was obtained from the parent. This study was conducted in accordance with the Declaration of Helsinki [28] and approved by the Ethics Committee of the NCNP, Japan (A2012–091, A2019-092).

### 2.2 Fluorescence immunohistochemistry

CBA was performed with antibodies against NMDAR, AMPAR, LGI1, DPPX, Caspr2, and GABABR using the “Autoimmune Encephalitis Mosaic 6 indirect immunofluorescence technique (IIFT) test kit (FA 112d-1010-6, Lubeck, Germany).” NMDAR was also tested using the “Autoimmune Encephalitis Mosaic 3 IIFT Test Kit (FA 111m-1010-3; Lubeck, Germany)”.

The TBA was performed using the rat cerebellum and hippocampus (Autoimmune Encephalitis Mosaic 3, IIFT test kit, FA 111m-1010-3, Lubeck, Germany). N.Y. and K.H. received EUROIMMUN Academy online training “Medical Laboratory Diagnosis” in 2021.

The assays were performed according to the manufacturer's instructions, with slight modifications. In the CSF analyses, the positive control (anti-NMDAR antibody) was diluted 3-fold with deionized water, whereas the samples were not diluted. For serum analyses, both positive controls and samples were diluted 10-fold with deionized water. The sample of 30 μL was applied to each cell or tissue spot, followed by incubation for 30 min. The slides were briefly washed with phosphate-buffered saline (PBS) containing Tween (PBS-T) and then immersed in PBS-T for 5 min. Twenty-five μL of fluorescein-labeled goat anti-human globulin was added to each spot, and incubated for 30 min. The slides were washed briefly with PBS-T followed by the incubation with PBS-T for 5 min, then embedded with 10 μL embedding medium.

The images used for the analysis were automatically merged from the original 35 images at 10 × magnification using an All-in-one Fluorescence Microscope (BZ-X800, Keyence, Osaka, Japan). The signal intensity in the cells was automatically calculated using the Hybrid Cell Counting Application (BZ-H4C, Keyence, Osaka, Japan) with the BZ-X Analyzer software (BZ-H4A, Keyence) (Supplementary Fig. 1).

NMDAR staining intensities were analyzed using a Hybrid Cell Counting Application (BZ-H4C Analyzer; Keyence) and NMDAR-positive cells were automatically counted in the field of each slide. The signal intensity of the CSF-stained images of patients with schizophrenia and controls was quantified, and the cut-off values were determined from the results of the positive controls (18 samples for mosaic 6 and 38 samples for mosaic 3). The averages of the cut-off values were calculated, which we termed as “strict criteria.” The samples were determined to be NMDAR-positive or weakly positive when signals were detected in the NMDAR field but not in the control cell field. When the sample was positive or weakly positive, the experiment was repeated and a consensus assessment was made. Samples were categorized as “control cell positive,” if signals were detected in the control cell field. Imaging data were sent to EUROIMMUN Inc. for a final assessment to confirm positivity.

### 2.3 Immunoblotting

CSF samples were tested using immunoblotting kit of EUROLINE PNS 12 Ag (DL 1111-1601-7 G; Euroimmun, Lübeck, Germany) and EUROLINE antinuclear antibody (ANA) Profile 23 (DL 1590-1601-23G; Euroimmun, Lübeck, Germany). The PNS kit was used for detection of human autoantibodies of Tr (delta/notch-like epidermal growth factor-related receptor: DNER), glutamic acid decarboxylase (GAD65), zinc finger protein 4 (Zic4), Titin, SRY-related HMG-Box Gene 1 (SOX1), Recoverin, neuronal nuclear antibody type 1 (Hu), Purkinje cell cytoplasm (Yo), neuronal nuclear antibody type 2 (Ri), paraneoplastic antigen (PNMA) Ma/Ta, CV2, and Amphiphysin. The ANA kit was used for detection of human autoantibodies of double-strand DNA, nucleosomes, histones, Sjogren syndrome antigen (SS-A, SS-B), Ro/SS-A 52 kDa (Ro-52), Smith antigen (Sm), nuclear ribonucleoprotein (nRNP)/Sm, Mi-2α, Mi-2β, ku, centromere protein (CENP-A, CENP-B), Sp100 nuclear antigen (Sp100), promyelocytic leukemia protein (PML), DNA topoisomerase Ⅰ (Scl-70), polymyositis-scleroderma overlap syndrome-100 (PM-Scl-100), PM-Scl-75, retinitis pigmentosa 11 (RP11), RP155, glycoprotein 210 (gp210), proliferating cell nuclear antigen (PCNA) and dense fine speckles 70 (DFS70). The assay was performed according to the manufacturer's instructions with slight modifications. The amount of CSF and positive control used was reduced from 1 mL to 100 μL. The test strips were first activated with the sample buffer attached to the kits and then incubated with the samples. A 100 μL CSF sample was overlaid on the test strip and covered with parafilm onto the test strip to prevent desiccation. The Parafilm was peeled off and recovered every 30 min to agitate the sample.

### 2.4 Statistical analysis

Age and sex differences were compared using the unpaired *t*-test and chi-square test. Chi-squared and Fisher's exact tests were used to assess whether the number of positive cases differed between patients with schizophrenia and controls. Statistical significance was set at p < 0.05. All statistical analyses were performed using SPSS (ver29, IBM Inc., NY, USA).

## 3 Results

**Fig. 1:**
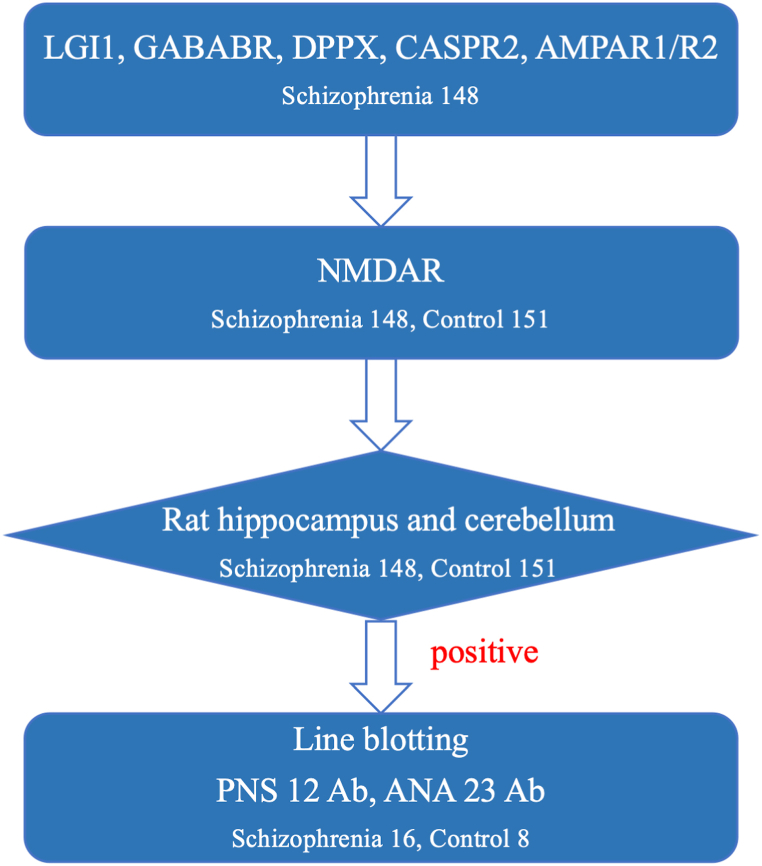
Flowchart of the experiments.

A flowchart of the experiment is shown in Fig. 1. Although not all the patients underwent MRI and EEG tests, all patients were ruled out for possible or probable autoimmune encephalitis based on the diagnostic criteria for autoimmune encephalitis [9] in their clinical course and were finally diagnosed with schizophrenia. Demographic and clinical data of the participants are presented in Table 1. The mean duration of the disease was 13.2 years. There were no significant differences in age or sex between the schizophrenia and control groups.

**Table 1:** Demographic and clinical data of participants.

	Schizophrenia (n = 148)	Control (n = 151)	Comparison
Age years, mean ± SD (range)	39.0 ± 11.0 (15–65)	38.2 ± 12.5 (19–65)	p = 0.541 (*t*-test, df = 1)
Sex, Male (%)	88 (59.5 %)	60 (40.5 %)	p = 0.226 (χ^2^ test, df = 1)
duration of illness years mean ± SD (range)	13.2 ± 9.95 (0–45)	–	–
Antipsychotic use (CP equivalent^a^) mg/day	400	–	–
Total PANSS scores mean (range)	65.0 (33–129)	–	–

SD, standard deviation; PANSS, Positive and Negative Syndrome Scale; df, degrees of freedom.

a CP equivalent: chlorpromazine equivalent.

First, the most frequent antibodies against autoimmune encephalitis, including AMPAR, LGI1, DPPX, Caspr2, and GABABR, were screened in 148 CSF samples from patients with schizophrenia using CBA. All samples were distinctly negative for these antibodies.

**Fig. 2:**
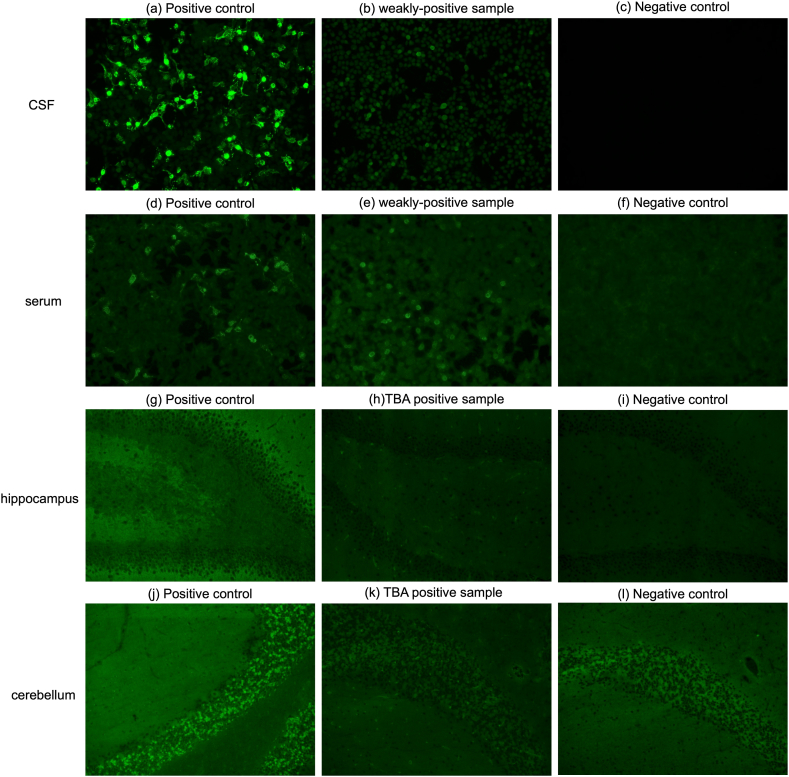
(a)–(f): Fluorescent images of NMDAR cell-based assay in CSF (a)–(c) and serum (d)–(f). The middle images of the CSF (b) and serum (e) are originated from identical participant. (g)–(l): Fluorescent images of tissue-based assay in the rat hippocampus (g)–(i) and the rat cerebellum (j)–(l).

Next, we analyzed NMDAR antibodies in CSF samples from 148 patients with schizophrenia and 151 controls using CBA. Although none of those samples was ‘positive’ using strict criteria that were compatible with the positive controls, we found five ‘weakly positive’ samples (two patients with schizophrenia and three controls) that had higher signal intensity in the NMDAR field compared to the control cell field (Fig. 2[a-c]), and the findings were confirmed in duplicate experiments. For positive/negative determination, we sent the images of a schizophrenia sample that had the highest signal intensity, to EUROIMMUN Inc., and their assessment was ‘negative’ following their criteria, suggesting that our “weakly positive” samples were all negative. We also evaluated the simultaneous serum samples of the above five CSF ‘weakly positive’ cases using CBA. We found that one schizophrenia sample also showed ‘weakly positive’ in serum (Fig. 2[d-f]). However, according to EUROIMMUN Inc., all serum samples tested negative.

Thereafter, TBA was performed to screen for other anti-neuronal antibodies. We detected positive signals in the CSF samples from 13 patients with schizophrenia and eight controls. The demographic data of the positive cases and the immunoblotting patterns of TBA are shown in Table 2. Representative images of TBA-positive samples are shown in Fig. 2[g-l]. The chi-square test did not detect a significant difference in the tissue-positive ratio between the schizophrenia and control groups. Among these samples, 11 schizophrenia and seven control samples showed positive signals in CBA control cells.

**Table 2:** Demographic and clinical data of the tissue-positive participants.

Case	Age	Sex	Diagnosis	Control cell	ANA	Immunofluorescence pattern
1	26	female	Schizophrenia	＋	DFS70	distribution of the speckles in the nuclei
2	34	female	Schizophrenia	＋	DFS70	distribution of the speckles in the nuclei
3	33	male	Schizophrenia	＋	DFS70	distribution of the speckles in the nuclei
*4	35	female	Schizophrenia	＋	DFS70	distribution of the speckles in the nuclei
5	31	male	Schizophrenia	＋	DFS70	distribution of the speckles in the nuclei
6	31	male	Schizophrenia	＋	DFS70	distribution of the speckles in the nuclei
*7	64	female	Schizophrenia	＋	DFS70	distribution of the speckles in the nuclei
*8	36	male	Schizophrenia	＋	DFS70	distribution of the speckles in the nuclei
9	59	male	Schizophrenia	＋	CENP A, CENP B	coarse speckles and aligned at the chromatin mass
10	57	female	Schizophrenia	＋	–	Granular fluorescence of almost all neuronal nuclei
11	28	male	Schizophrenia	＋	DFS70	distribution of the speckles in the nuclei
*12	37	female	Schizophrenia	–	–	weak neuro-endothelium like fluorescence
13	65	female	Schizophrenia	–	–	weak neuro-endothelium like fluorescence
1	42	female	Control	＋	DFS70	distribution of the speckles in the nuclei
2	49	female	Control	＋	DFS70	distribution of the speckles in the nuclei
3	22	female	Control	＋	DFS70	distribution of the speckles in the nuclei
4	56	male	Control	＋	DFS70	distribution of the speckles in the nuclei
5	31	male	Control	＋	DFS70	distribution of the speckles in the nuclei
6	19	male	Control	＋	DFS70	distribution of the speckles in the nuclei
7	60	male	Control	＋	–	Fluorescence of all cell nuclei
8	59	male	Control	–	–	Fluorescence of all cell nuclei. Cytoplasmic fluorescence of the Purkinje cells

ANA, antinuclear antibody; DFS, dense fine speckles; CENP-A, centromere protein A; CENP-B, centromere protein B.

**Fig. 3:**
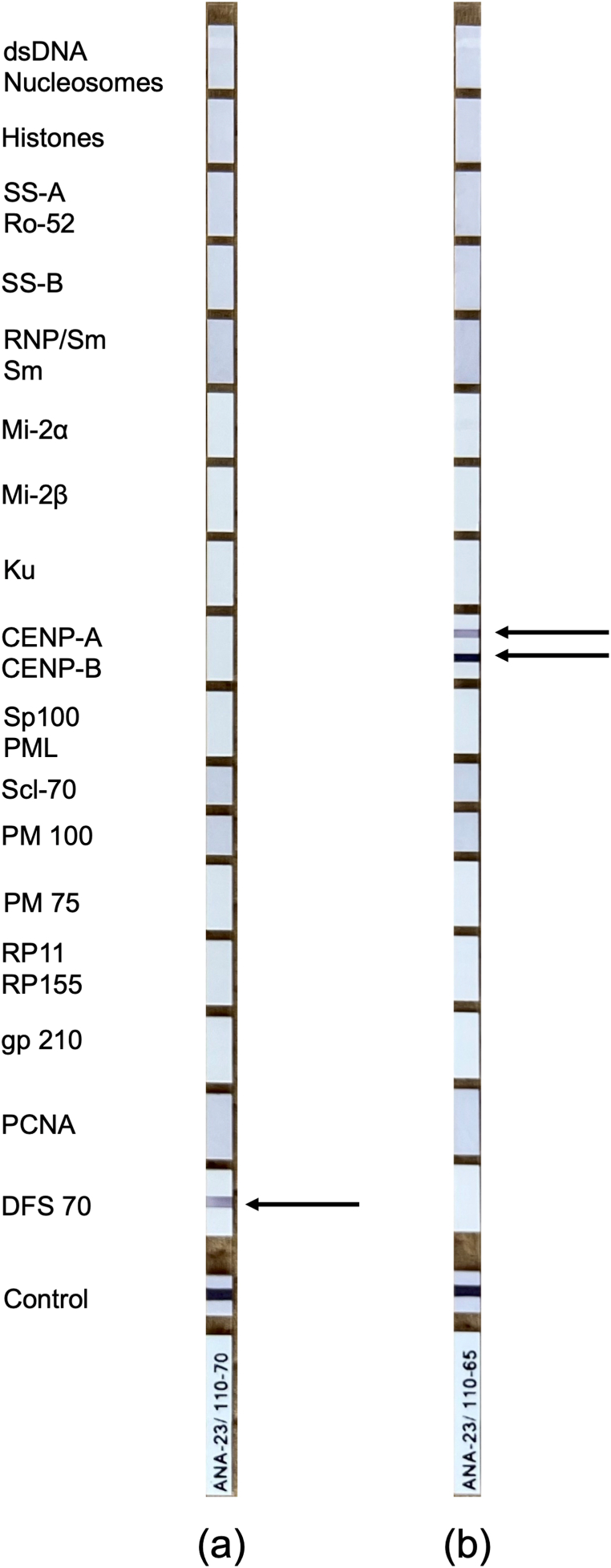
Images of ANA Immunoblotting of (a)DFS70 positive, (b)CENP-A and CENP-B positive. dsDNA, double-strand DNA; SS-A, Sjogren syndrome antigen A; Ro-52, Ro/SS-A 52 kDa; SS-B, Sjogren syndrome antigen B; nRNP/Sm, nuclear ribonucleoprotein; Sm, Smith antigen; CENP-A, centromere protein A; CENP-B, centromere protein B; Sp100, Sp100 nuclear antigen; PML, promyelocytic leukemia protein; Scl-70, DNA topoisomerase Ⅰ; PM 100, polymyositis-scleroderma overlap syndrome-100; PM 75, polymyositis-scleroderma overlap syndrome-75; RP11, retinitis pigmentosa 11; RP155, retinitis pigmentosa 155; gp210, glycoprotein 210; PCNA, proliferating cell nuclear antigen and DFS70, dense fine speckles 70.

To identify the antibodies in TBA-positive samples, immunoblotting was performed for 12 PNS antibodies and 23 ANAs. None of the samples tested positive for any of the 12 PNS antibodies. Nine patients with schizophrenia and six control controls tested positive for DFS70 antibodies (Fig. 3[a]). All of these DFS70-positive samples were positive for CBA in the control cells. We also found that one patient sample was positive for CENP-A and CENP-B (Fig. 3[b]), whereas no antibodies other than DFS70 were detected in the controls. Thus, among the TBA-positive samples, three schizophrenia samples and two control samples remained unspecified for their antibodies.

We examined the clinical characteristics of 13 patients with TBA-positive schizophrenia who underwent immunoblotting. As a result, clinical information was available for 12 individuals, of which 5 met the criteria for possible autoimmune psychosis according to Pollak et al., [29]. The demographic profile of patients who met the criteria of possible autoimmune psychosis is shown in Table 3.

**Table 3:** Demographic profile of schizophrenia patients who met the criteria of possible autoimmune psychosis.

Case	Age	Sex	ANA	onset <3 m with	CSF pleocytosis	MRI	EEG	other
4	35	female	DFS70	fever adverse response to antipsychotic	–	non-specific T2/FLAIR high lesion	–	
7	64	female	DFS70	cognitive dysfunction	–	–	–	
8	36	male	DFS70	cognitive dysfunction	–	–	–	low DDK
12	37	female	–	cognitive dysfunction	–	non-specific T2/FLAIR high lesion	NA	OGB － IgG index －

## 4 Discussion

In the present study, we examined anti-neuronal autoantibodies related to psychosis using CSF samples from patients with schizophrenia and controls matched for age and sex. The analyzed anti-neuronal autoantibodies, including anti-NMDAR antibodies, tested negative for schizophrenia. In the tissue-based analyses, we detected positive signals in the CSFs of 13 patients with schizophrenia and eight controls, although the positive rates were not significantly different between the diagnostic groups. Among these positive samples, eight schizophrenia and six control samples were positive for DFS70, as determined using immunoblotting.

Several studies have attempted to detect NMDAR in patients experiencing the first episode of psychosis or in the acute phase of schizophrenia. In the studies analyzing blood samples, 2–10 % of the patients with schizophrenia were reported to be positive for NMDAR antibody [[30], [31], [32]]. In contrast, in studies using the CSF, the antibody was barely detected in patients with schizophrenia [19,33]. Our results using the CSF of patients with chronic schizophrenia are consistent with those of subsequent CSF studies.

In our study, several samples from patients with schizophrenia were weakly positive for NMDAR, and one serum sample of the CSF ‘weakly positive’ schizophrenia was also weakly positive for NMDAR, although they were all negative according to strict criteria. However, the clinical significance of these findings remained unclear. Since there were no differences in the incidence of positivity between the schizophrenia and control groups, it is unlikely that subthreshold antibodies had a significant effect on this disorder. Other antibodies, including those against LGI1, GABABR, DPPX, AMPAR1/R2, and CASPR2, were absent from the CSFs of patients with schizophrenia. Thus, our results suggest that NMDAR and the most frequent PNS antibodies are unlikely to be involved in the pathophysiology of schizophrenia, at least in patients in the chronic state.

For TBA, we detected positive signals in the CSFs of 13 patients with schizophrenia and eight controls. Among these samples, the CSFs of nine patients with schizophrenia and six controls were positive for DFS70 antibodies. DFS70 is an antinuclear antibody that is associated with atopic dermatitis and asthma [34,35]. Although DFS70 has also been detected in healthy individuals, its pathogenic significance remains unclear [36]. Taken together, our results suggest that DFS70 is unlikely to play a major role in the pathogenesis of schizophrenia, as DFS70 positivity is comparable between the schizophrenia and control groups. Alternatively, the high proportion of DFS70 in both groups may have impaired the validity of tissue-based screening tests and impeded autoantibody detection. Because 71 % of TBA-positive cases were DFS70-positive, one should be cautious of the presence of DFS70 when detecting antibodies in tissue samples; DFS70 antibodies might need to be removed before further analyses. Additionally, all DFS70-positive cases were also positive for control cells, suggesting that DFS70 positivity should be suspected, at least in samples that are positive for control cells.

Unknown antigen-antibody reactions were found in three schizophrenia samples and two controls. Since the control samples were also positive, and there was no significant difference in the positive ratio between the two groups, it is unlikely that the unspecified antibodies were the primary cause of schizophrenia. Overinterpretation of nonspecific autoantibodies has been reported to be a frequent contributor to the misdiagnosis of autoimmune encephalitis [37] and caution should be exercised when researching mental disorders.

A previous report suggested that DFS70-positive females have a higher rate of concomitant autoantibodies, whereas DFS70-positive males do not, suggesting that DFS70 is a useful biomarker for the absence of other autoantibodies in male [38]. Therefore, the possibility of the concomitant presence of autoantibodies in some DFS70-positive patients cannot be ruled out.

Additionally, novel autoantibodies have been identified in schizophrenia in recent years [6,39], and the possibility of the presence of specific autoantibodies related to schizophrenia below the TBA threshold cannot be ruled out. In our study, four out of 12 TBA-positive schizophrenia participants met the criteria for possible autoimmune psychosis, and there remains the possibility that the four patients had psychogenic antibodies. In addition, we cannot deny the possibility that the autoantibodies are specific to human tissue and are not detected in the rodent brain and/or that autoantibodies are vulnerable to fixation.

Most of the samples were obtained from patients with chronic schizophrenia in a relatively stable state; therefore, we cannot deny the possibility that the analyzed antibodies were elevated in the acute phase but decreased below the threshold at the time of CSF collection. In addition, we analyzed only a limited number of antibodies using high-sensitivity CBA. Therefore, the existence of antibodies with low titers, which are difficult to detect using TBA, could not be ruled out.

## 5 Conclusions

Our study indicates that autoantibodies, particularly those targeting NMDAR and major PNS antibodies, are rarely found in the CSFs of patients with chronic schizophrenia. Furthermore, we emphasize the importance of considering the impact of anti-DFS70 antibodies when conducting tissue-based autoantibody screening using CSF. Overall, we found no evidence that neuronal autoantibodies in the CSF play a role in the pathogenesis of chronic schizophrenia.

## Ethical Statement

This study was approved by the Ethics Committee of the NCNP, Japan (A2012-091: June 23rd, 2021), (A2019-092: March 16th, 2020). For participants under the age of 20, informed assent was obtained from the individual after which written informed consent was obtained from the parent.

## Funding

This research was supported by 10.13039/100009619AMED under Grant Number JP20ak0101151 (K·H.).

## Data availability statement

The data will be accessed in the database under construction (tentative name; IDID). Until then we will provide data upon request.

## CRediT authorship contribution statement

**Takako Enokida:** Writing – original draft, Resources, Investigation, Formal analysis. **Nanako Yoshida:** Writing – original draft, Methodology, Investigation, Formal analysis. **Megumi Tatsumi:** Resources. **Shinsuke Hidese:** Resources. **Yu-ichi Goto:** Supervision. **Mikio Hoshino:** Supervision. **Hiroshi Kunugi:** Writing – review & editing. **Kotaro Hattori:** Writing – original draft, Resources, Methodology, Funding acquisition, Formal analysis, Conceptualization.

## Declaration of competing interest

The authors declare that they have no known competing financial interests or personal relationships that could have appeared to influence the work reported in this paper.

## Figures and Tables

**figs1:**